# Home based postpartum care and determinants in Ethiopia: A multilevel analysis

**DOI:** 10.1371/journal.pone.0272489

**Published:** 2022-08-25

**Authors:** Binyam Minuye Birhane, Wubet Alebachew Bayih, Muluken Chanie, Getaneh Awoke, Amare Simegn, Sintayehu Asnakew, Melkalem Mamuye, Abebaw Yeshambel, Tewachew Muche, Asmamaw Demis, Tigabu Munye, Aklilu Endalamaw, Yeshambew Eshetie, Demewoz Kefale, Ermias Sisay Chanie, Zemen Mengesha Yalew, Demeke Mesfin Belay

**Affiliations:** 1 College Health Sciences, Debre Tabor University, Debre Tabor, Ethiopia; 2 Debre Tabor Health Science College, Debre Tabor, Ethiopia; 3 College of Health Sciences, Woldia University, Ethiopia; 4 School of Health Sciences, College Medicine and Health Sciences, Bahirdar University, Bahirdar, Ethiopia; 5 School of Public Health, The University of Queensland, Brisbane, Australia; 6 Department Comprehensive Nursing, College of Health Sciences, Wollo University, Ethiopia; PLOS, UNITED KINGDOM

## Abstract

**Introduction:**

Neonatal mortality remains a persisting public health challenge in Ethiopia. Timely intervention to neonatal morbidity and early neonatal care visit could reduce the burden of mortality. Studies related to home based postnatal care is limited in Ethiopia. Therefore, this study aimed to assess home based postnatal care visits and determinants in Ethiopia.

**Methods:**

A secondary data analysis using 2016 EDHS data was conducted among 7590 women who had live births two years preceding the survey. A multilevel mixed-effect logistic regression analysis model was used and those variables with a P-value ≤ of 0.05 in multivariable analysis were considered as predictors. Results: Home based postpartum care by health care providers was 6.3% and 67.9% of women gave birth at home. Women perceived that distance is not big problem [AOR = 1.37; 95% CI: 1.06, 1.68], richer wealth index [AOR = 1.69; 95% CI: 1.15, 2.48], attending antenatal care visit [AOR = 2.17; 95% CI:1.57, 2.99], giving birth in health institution [AOR = 2.07; 95% CI:1.53, 2.80], giving birth by cesarean section [AOR = 3.41; 95% CI: 2.33, 4.99], and having awareness about neonatal danger sign [AOR = 3.68; 95% CI: 2.90,4.70] were factors associated with home based postpartum care.

**Conclusion:**

Home based care by health care providers was low. Therefore, measures should be taken in increasing the number of nearby health care facility, strengthen the continuum of care on antenatal care follow-up, institutional delivery and improve mother’s knowledge about neonatal danger sign.

## Introduction

Globally, 2.4 million neonates died within the first month of life; of which 6,700 neonatal deaths happen every day. Many preventable causes attribute to neonatal death; preterm birth complications, birth asphyxia, congenital anomalies, diarrhea, and malaria were some of the predictors. Higher neonatal mortality was in sub-Saharan Africa and South Asia with a mortality rate of 25 and 27 death per 1,000 live births, respectively [1–3]. Specifically, in Ethiopia, the neonatal mortality rate was 30 deaths per 1,000 live births [4]. Effective interventions like postnatal home-based care and others could prevent two-thirds of neonatal mortality [5].

Home-based neonatal care visit by health care providers is one of the main strategies in reducing neonatal mortality and morbidity [6–8]. Thus, home-based neonatal care visits lower the neonatal mortality rate by 13% to 34% [9–12]. One study showed that death was 84% less likely among neonates who received home-based care [13].The same is true in Bangladesh, where neonatal mortality was 67% lower for neonates who received a home care visit on day one and 64% on day two [14].

Postnatal home visits are effective in improving neonatal care. These cares are: exclusive breastfeeding, skin to skin contact, identify and support additional care for newborns, increase awareness about neonatal danger signs, facilitate safe umbilical cord care, prepare the neonate for immunization, promote parenting skills, address a large segment of the rural community and improve health-seeking behaviors [12, 15–20]. Moreover, implementing home-based neonatal care is crucial for early detection and follow-up of neonates at high risk of long-term health and developmental impairments [21].

By 2035, all countries will have reached the target of 10 or fewer newborn deaths per 1,000 live births that they planned to reduce death and disability by ensuring that no newborn is left behind [22]. It can be achieved by improving community-based health care integrated interventions. Home visiting, counseling, timely recognition of danger signs, strengthen finances to arrange for transport and affordability of health care cost, and accessibility to a health facility [23, 24] are strategies that have been and planned to implement. Empowering women, families and communities, integrate maternal and newborn care, and support the mother-baby relationship [25] are the main strategies to reduce neonatal mortality.

World Health Organization (WHO) has planned four goals to eliminate preventable newborn and stillbirth deaths by 2025 targeted at 90% of mothers have four or more antenatal visits (ANC) and delivered by skill birth attendant, 80% of the mothers receive routine postnatal care within two days of birth and countries should have implementation plan in protecting small and sick newborns [26].

United Nations Children’s Fund (UNICEF) and WHO recommends at least two home visits by a skilled attendant during and immediately after birth irrespective of where the birth takes place. For home births, the first visit within 24 hours from birth and the second visit on day three, and the third visit by the end of the seventh week. For babies born in a health facility, the first home visit being as soon as the mother and baby reach home. The remaining visits as for home births [6, 27, 28].

Ineffective home-based neonatal care in Ethiopia had been related to workload, shortage of trained health care worker and budget at health posts, limited presence of health insurance, unable to notify their birth to health care providers (HCPs), limited home visit during pregnancy, poor supervision, inadequate drugs and supply, distance and topography, lack of attending a community meeting, poor ANC visit, and poor knowledge and belief [29–32]. Though there are improvements in health facility delivery, ANC visit [4], and integration of health extension workers in providing maternal and neonatal services [33], only 14.5% received PNC home visit within 3 days after birth and 12% to 24.1% within 42 days from health care providers [32, 34] in Ethiopia. Including these segregated data, studies about the magnitude and determinants of postnatal care visit were inconsistent and had inconclusive findings. Therefore, this study is helpful to estimate the overall magnitude and determinants of home based PNC in Ethiopia because the source is nationally represented EDHS data, which is helpful for policymakers to create, appraise, and advance additional preventive strategies.

## Methods

### Data source, study population and sampling technique

A community-based cross-sectional study was conducted in Ethiopia from 18 January to 27 June 2016. As per worldometer report on 22 April 2021, Ethiopia has a total population of 114,963,588 (20.9% lived in urban), total fertility was 4.2 live births per woman, the infant mortality rate was 29.5 per 1,000 live births, and under 5 mortality rate was 44 per 1,000 live births. In this country, there are nine regional states and two city administrations. Each region was stratified into urban and rural. A stratified two-stage cluster sampling technique was performed. Samples of Enumeration areas (EAs) were selected independently in each stratum in two stages. Firstly, a total of 645 EAs, 202 in urban areas and 443 in rural areas were selected with probability proportional to EAs size. The target group was all mothers who had a live birth in Ethiopia 2 years preceding the survey, and those mothers (15–49 years) in the selected enumeration areas were the study population [4]. Data was extracted from 7,590 postpartum mothers. Approval letter was obtained from the measure demographic and health survey (DHS), and the data set was downloaded from the DHS website (http://www.dhs program.com).

### Study variables

The outcome of interest was a home based postpartum care visit by health care providers (Medical doctor, Midwife, Nurse, Health Officer, Health Extension Workers, and Traditional Birth Attendants) among women who had live birth 2 years preceding the survey. This variable was dummy coded so that respondents who reported having postnatal home visit within the postpartum period, by health care providers coded as “Yes”, otherwise “No”. Socio-demographic, obstetric, and facility-related variables were included. Socio-demographic variables were age, residence, religion, marital status, educational status, and economic status. Obstetric-related variables were ANC, place of delivery, and mode of delivery. community level related variables were the mother’s perception of home distance from health facility categorized as “big problem” or “not a big problem”, and residence (urban, rural).

### Statistical analysis

Data extracted from 2016 EDHS data. Data cleaning, recoding and analysis were carried out using SPSS statistical software version 24. Postnatal care visit varies across each cluster. Sampling weight was applied for all analysis procedures to account for complex survey design and unequal probabilities of selection. Since, the data was national survey data, a hierarchical and cluster nature multilevel analysis model was used. The sampling weight of cluster sampling was adjusted using “Svy” command. The measure of community variation was estimated by the interclass coefficient (ICC). In the null model, ICC was 22.8% which showed that this amount of variation in home based postnatal care visit was attributed to community-level factors; indicating the multilevel model is better. Proportional change in variance (PCV) was estimated for each model with respect to the null model; PCV = (variance in null model-variance in the model used)/variance in the null model) to show how much variance explains the model.

The likelihood ratio test was determined to test the significance of the variance of random intercept. The analysis was conducted in four models, which are the Null model, model 1, model 2, and model 3 and model comparison was done using deviance. The Null model is a model without explanatory variable, a model I with individual-level variables), model II with community-level variable and model III with both individual and community-level factors. The best model was selected using Akaike information criterion (AIC) model selection criteria. The lowest value of AIC could be the best model (Table 1). Therefore, model 3 which is a full model consisting individual and community level variables were selected. Variables with p values ≤ of 0.05 in the multilevel analysis were considered statistically significant determinants. Finally, the result was presented using figures, tables and texts.

**Table 1:** Multilevel fixed effect model of individual and community level factors predicting postnatal care visit at home by health care professional in Ethiopia.

Random effect	Null model	Model I	Model II	Model III
Variance	0.971	0.473	0.471	0.354
ICC (%)	22.8%	12.6%	12.5%	9.7%
PCV (%)	Ref	51.2%	51.4%	63.5%
Median odd ratio (MOR)	2.55	1.92	1.91	1.76
Log likelihood	-1762.458	-1597.72	-1694.46	-1574.76
AIC	3528.9	3223.4	3416.9	3201.5

### Ethical considerations

Ethical approval was not obtained because of the data was extracted from EDHS 2016 data. Permission to use the EDHS data was received from measure DHS international program.

## Results

### Socio-demographic characteristics

A total of 7,590 women who gave live birth 2 years preceding the survey were included. Almost half (50.4%) of women were within the age group of 25–34 years, and 93.7% were married. The majority (87.2%) were from rural residency. Thirty -eight percent of women were orthodox in religion. Almost two-third (63.1%) of women were illiterate. More than half of women (58.1%) perceived the distance from nearby health facilities as a big problem for utilization of postpartum care (Table 2).

**Table 2:** Socio-demographic and economic characteristics of postpartum women in Ethiopia.

Variables	Weighted Frequency		Percentage (%)
Age			
15–24 years	1804		23.80
25–34 years	3827		50.40
≥35 years	1959		25.80
Residence			
Urban	969		12.80
Rural	6621		87.20
Marital status			
Unmarried	481		6.30
Married	7109		93.7
Religion			
Orthodox	2884		38
Muslim	2821		37.2
Protestant	1651		21.8
*Others	234		3.0
Educational status			
No education	4791		63.10
Primary education	2150		28.30
Secondary and above	649		8.60
Wealth index			
Poorest	1651		21.80
Poorer	1654		21.80
Middle	1588		20.90
Richer	1427		18.80
Richest	1269		16.70
Distance to Health facility			
Big problem	4407	58.10	
Not big problem	3183	41.90	

*Others-Catholic, tradition

### Postnatal care visit

**Fig 1:**
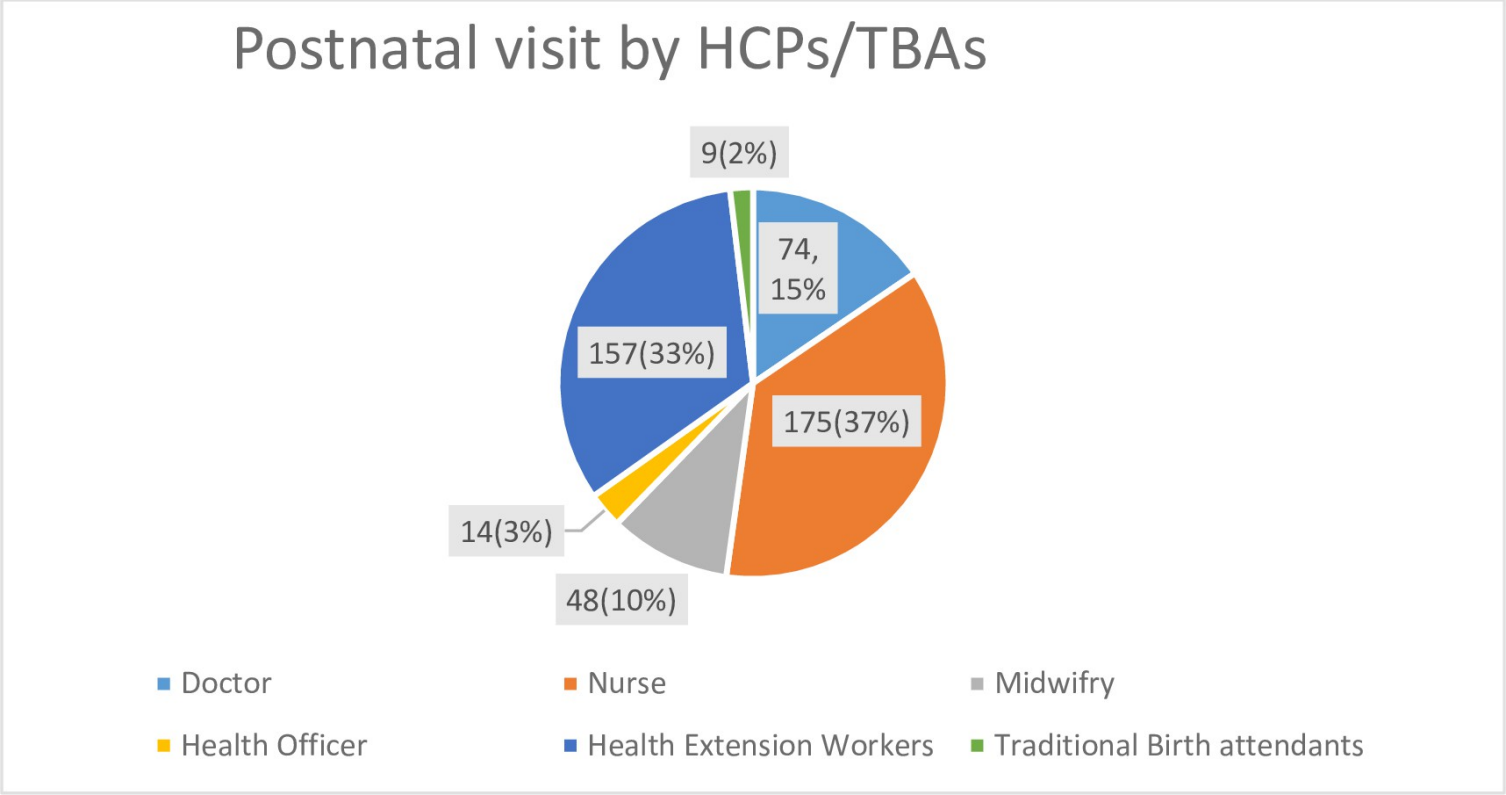
Postnatal care visit at home provided by health care providers, Ethiopia.

In the current study, home based postpartum care visit was 6.3% [95% CI; 6.19, 6.41]. The majority (37%) visit was conducted by nurses followed by health extension workers (33%) (Fig 1).

### Determinants of home based postpartum care

Regarding health care service utilization, 62.8% of women had ANC visit and more than two-third (67.9%) of women gave birth at home. The majority (97.6% and 95.7%) of women had a spontaneous vaginal delivery and no awareness of neonatal danger signs, respectively. In bi-variable mixed-effect logistic regression model; residence, perceived distance from health facility, educational status, wealth index, antenatal care visit, place of delivery, practice of skin-to-skin contact, mode of delivery, awareness of neonatal danger sign, region, and owns mobile phone were significantly associated with postpartum home visit. Whereas in multivariable mixed-effect logistic regression model (model III); wealth index, perceived distance from health facility, place of delivery, mode of delivery, ANC visit, and awareness of neonatal danger were independently associated with home based care (Table 3). Full model (model 3) was the best model to identify predictors of postnatal care visit since AIC is the lowest in model III. As such, the odds of home based postnatal care visit among women who perceived distance from health care institutions is not a big problem were 1.37 times more likely than its counterparts [AOR = 1.37; 95% CI:1.06,1.68]. Similarly, newborns from better socio-economic status were about 1.69 times more likely to have home based postnatal visit than from low socio-economic status [AOR = 1.69; 95% CI: 1.15, 2.48]. Other factors such as having ANC follow up [AOR = 2.17; 95% CI:1.57, 2.99], institutional delivery [AOR = 2.07; 95% CI:1.53, 2.80], delivery by cesarean section (C/S) [AOR = 3.41; 95% CI: 2.33, 4.99], awareness on neonatal danger sign [AOR = 3.68; 95% CI: 2.90,4.70], and women from Tigray region [AOR = 2.82; 95% CI:1.75,4.54] were associated with postnatal home care visits (Table 3).

**Table 3:** Determinants of home based postpartum care by health care providers among postpartum women in Ethiopia.

Variable	Null model	Model I	Model II	Model III
Residence Urban				
Rural			1.47 (1.08, 2.01)	0.87(0.56,1.34)
			Ref	Ref
Educational status No education		Ref		Ref
Primary education Secondary and above		1.03 (0.804,1.31)		1.04 (0.81,1.33)
		1.22 (0.87,1.71)		1.27 (0.90,1.79)
Wealth index				
** **Poorest		Ref		Ref
** **Poorer		1.35 (0.95,1.91)		1.29 (0.89,1.85)
** **Middle		1.42 (0.99, 2.02)		1.36 (0.94,1.97)
** **Richer		1.71 (1.19,2.44)		1.69 (1.15, 2.48) **
** **Richest		1.26 (0.86,1.84)		1.26(0.77,2.04)
Distance to Health facility				
** **Big problem			Ref	Ref
** **Not big problem			1.55 (1.24,1.93)	1.37 (1.06,1.68) **
ANC Visit				
** **Yes		2.45 (1.78, 3.37)		2.17(1.57, 2.99) **
** **NO		Ref		Ref
Awareness on neonatal danger sign				
** **Yes		4.04 (3.18, 5.13)		3.68 (2.90,4.70) **
** **No		Ref		Ref
Place of delivery				
** **Health institution		1.87 (1.39, 2.52)		2.07(1.53, 2.80) **
** **Home		Ref		Ref
Mode of delivery				
** **Caesarean section		3.35 (2.30, 4.88)		3.41 (2.33, 4.99) **
** **Vaginal		Ref		Ref
Own mobile phone				
** **Yes		1.20 (0.91,1.60)		1.27 (0.95,1.70)
** **No		Ref		Ref
Skin to skin contact				
Yes		0.85 (0.66,1.09)		0.85 (0.66,1.09)
** **No		Ref		Ref
Region				
Oromia			Ref	Ref
Tigray			4.30 (2.68, 6.88)	2.82 (1.75,4.54) **
Afar			1.13 (0.64, 2.02)	1.63 (0.89, 2.99)
Amhara			1.89 (1.14, 3.12)	1.52 (0.91, 2.48)
Somali			0.67 (0.36, 1.21)	0.89 (0.48,1.66)
Benishangul-			2.06 (1.21, 3.52)	1.63 (0.96,2.78)
Gumuz			1.71 (1.04, 2.81)	1.32 (0.81,2.15)
SNNPR			0.59 (0.29, 1.18)	0.69 (0.34, 1.39)
Gambela			1.93 (1.08, 3.45)	1.52 (0.85,2.71)
Harari			2.51 (1.39, 4.51)	1.37 (0.76, 2.48)
Addis Ababa			1.94 (1.07, 3.53)	1.61 (0.89,2.92)
Dire Dawa				

Significant at

** P-value<0.05; SNNPR-Southern nation and nationalities of Ethiopia

## Discussion

Postnatal care visit is one of the strategies to reduce neonatal morbidity and mortality. The current study showed that Wealth index, perceived distance from health facility, place of delivery, ANC visit, mode of delivery, and awareness of neonatal danger sign were independently associated with home based postnatal care visit.

In the current study, home based postnatal care visit was 6.3%. This study is consistent with the study conducted in Ethiopia 7% [35]. This is justified that the low level of home-based neonatal care could be related to challenges that health extension workers faced like productivity and efficiency, working and living conditions, workload, the capacity of health posts, and poor supervision [36]. But, higher than the study conducted in Ethiopia, 2.9% [37]. The possible reason for the discrepancy could be the current study includes both institutional and home deliveries, but the previous study includes only home deliveries.

The current study is lower than the studies conducted in southern Ethiopia (12.4%) [34], Northern Ethiopia (14.5%) [32], and Pakistan (25%) [10]. The difference could be the current study targets women who gave birth in the last 2 years preceding the survey with a large sample size which may have recall bias, whereas the study conducted in southern Ethiopia targets women who had live birth less than 6months of age.

The odds of postnatal home visit among women who perceived distance from health care institutions is not a big problem were more likely than women perceived distance is a big problem. Previous studies suggested that the closer the health institution the better to utilize the service [38, 39]. Evidence from Ethiopia also showed that more than 90% lived 1.5 or more hours from the health institution [40].

Women who were in the highest wealth index increase the odds of postnatal visit than women from low socioeconomic status. This is supported by studies conducted in Tanzania [41], Sri Lanka [42], and India [43]. Income has an effect on women’s autonomy to seek care [44, 45]. Moreover, living in low socioeconomic status is associated with home delivery [39]. Women who had better income could have better health care-seeking behaviors during pregnancy, labor and delivery. Moreover, they can access health care providers easily, have their own doctor and follow-up in the private clinic. One study in Ethiopia showed that 18% of women from the better income had postnatal care as compared to women only 8% from the poorest households had had PNC [46].

Antenatal care visit and birth at health care institutions increase the odds of home-based neonatal care. This is incongruent with other studies [31, 32, 47, 48]. The rationales could be facility delivery was associated with the practice of neonatal care interventions [49]. Mothers who deliver at health institutions were more aware of the need to have a follow-up visit, the schedule, importance of the postnatal visit and birth notification to health extension workers.

Women who deliver by cesarean section were more likely to have home-based neonatal care than women deliver by vagina. This is supported by prior studies in Ethiopia [35, 50–52].This might be due to women delivered by C/S are more likely to experience pain/discomfort [53] in the post-partum period; which in turn increase the need of assistance health care professionals, and also had better awareness on neonatal danger sign. In addition, mothers delivered by C/S faced more difficulty in initiating early breastfeeding [52].

Those mothers who had awareness on neonatal danger sign have better utilization of home base neonatal care. This is consistent with the study conducted in Uganda [54], which showed immediate breastfeeding after birth and exclusive breastfeeding were significantly higher among women who had home-based care (72.6%). Moreover, skin-to-skin contact helps the baby’s body self-regulate, initiate breast feeding, increase bonding, decrease maternal postpartum depressive symptoms, and increase sleep quality [55–57]. Those mothers who had better knowledge of neonatal danger signs had better birth preparedness and complication readiness [58–60] and decrease home birth [61]. This implies the government of Ethiopia should work harder on improving home based postnatal care visit by health care providers in reducing neonatal mortality and morbidity.

## Conclusion

Postnatal care visit at home by HCWs was low. Wealth index, perceived distance from the health facility, place of delivery, mode of delivery, ANC visit, and awareness of neonatal danger sign were associated with home based postnatal care. The finding has several implications for the health care system. Firstly, health care facilities should work harder to reduce the home birth and increase health care facilities. Secondly, home-based neonatal care by health care professionals should be encouraged to reduce neonatal morbidity and mortality. Therefore, measures should be taken in increasing the number of nearby health care facilities, strengthen the continuum of care on antenatal care follow-up, institutional delivery and improve mother’s knowledge of neonatal danger sign could bring the significant contribution in improving postnatal home visit by health care workers. The strength of the study was; the data was a national-based survey and with a large sample size. However, the study has its own limitation; does not show cause-effect relationship and variable which could be important to the outcome were missed.

## Supporting information

S1 File (PDF)

## Abbreviations

AIC: Akaike Information Criterion
ANC: Antenatal care Visit
AOR: Adjusted Odd Ratio
CI: Confidence Interval
C/S: caesarean section
DHS: Demographic and Health Survey
EA: Enumeration areas
EDHS: Ethiopian Demographic and Health Survey
HEWs: Health Extension Workers
ICC: Inter Class Coefficient
MOR: Median Odd Ratio
PNC: Postnatal Visit
SDG: Sustainable development goal
UNICEF: United Nations Children’s Fund
WHO: World Health Organization
